# A multiscale study of fungal endophyte communities of the foliar endosphere of native rubber trees in Eastern Amazon

**DOI:** 10.1038/s41598-018-34619-w

**Published:** 2018-11-01

**Authors:** Aline B. M. Vaz, Paula L. C. Fonseca, Fernanda Badotti, Demetra Skaltsas, Luiz M. R. Tomé, Allefi C. Silva, Mayara C. Cunha, Marco A. Soares, Vera L. Santos, Guilherme Oliveira, Priscilla Chaverri, Aristóteles Góes-Neto

**Affiliations:** 1 Department of Microbiology, Institute of Biological Sciences, Federal University of Minas Gerais (UFMG), Belo Horizonte, MG 31270-901 Brazil; 2 Faculdade de Minas (FAMINAS), Belo Horizonte, MG 31744-007 Brazil; 3 Department of Chemistry, Centro Federal de Educação Tecnológica de Minas Gerais (CEFET-MG), Belo Horizonte, MG 30480-000 Brazil; 4 Instituto Tecnológico Vale, Belém, PA 66055-090 Brazil; 5 Department of Plant Science and Landscape Architecture, University of Maryland, College Park, MD 20742 USA; 6 Escuela de Biología, Universidad de Costa Rica, San Pedro, San José Costa Rica

## Abstract

*Hevea brasiliensis* is a native hyperdiverse tree species in the Amazon basin with great economic importance since it produces the highest quality natural rubber. *H*. *brasiliensis*, in its natural habitat, may harbor fungal endophytes that help defend against phytopathogenic fungi. In this work, we investigated the fungal endophytic communities in two pristine areas in Eastern Amazon (Anavilhanas National Park – ANP and Caxiuanã National Forest – CNF) at different spatial scales: regional, local, individual (tree), and intra-individual (leaflet). Using a culture-based approach, 210 fungal endophytes were isolated from 240 sampling units and assigned to 46 distinct MOTUs based on sequencing of the nrITS DNA. The community compositions of the endophytomes are different at both regional and local scales, dominated by very few taxa and highly skewed toward rare taxa, with many endophytes infrequently isolated across hosts in sampled space. *Colletotrichum* sp. 1, a probably latent pathogen, was the most abundant endophytic putative species and was obtained from all individual host trees in both study areas. Although the second most abundant putative species differed between the two collection sites, *Clonostachys* sp. 1 and *Trichoderma* sp. 1, they are phylogenetically related (Hypocreales) mycoparasites. Thus, they probably exhibit the same ecological function in the foliar endosphere of rubber tree as antagonists of its fungal pathogens.

## Introduction

Fungal endophytes are defined functionally by their occurrence within asymptomatic tissues of plants without causing any apparent symptom of disease on the host plants^1,2^. They are internal colonizers of aboveground tissues in all plant species studied to date^3^. Fungal endophytes of woody plants are mainly horizontally transmitted, as evidenced by their scarcity in tree seeds^4,5^. Horizontal transmission occurs preferentially by spores and/or hyphal fragmentation from senescent plant tissues^6^, and these propagules may be disseminated by herbivores or by abiotic agents such as wind or rain^7^.

Assembly of local communities occurs by the sequential and repeated immigration of species from the regional species pool^8^. The mechanisms underlying species coexistence within fungal community assembly are a dichotomy of deterministic and stochastic processes. They are used to support the niche and neutral theories separately as well as their synthesis^9^. The niche theory suggests that differences in species ecological traits determine which species will establish in a niche space^10^. On the other hand, the neutral theory posits that species composition is related to stochastic events or is shaped by geographic distance, considering the neutrality in traits^11^.

Community composition of fungal endophytes is usually dependent on the geographic scale analyzed^3,12,13^. Geographic distance reflects environmental differences, and these differences may serve as ecological “filters” which selects for taxa that are better adapted to local conditions^12,14^. Ecological “filters” mediate the community assembly through processes of habitat filtering and species interactions^15,16^, and include both abiotic variables (e.g. temperature, UV exposure, precipitation) and biotic variables related to plant traits (e.g. biochemical defenses, tissue lignification)^15^.

Recently, a novel framework for examining fungal endophyte biology was proposed. The so-called dual-axis framework is based in two core axes: (i) mode of host-to-host transmission and (ii) degree of specificity to a particular host species or clade^17^. Conversely to previous classification systems^7^, this theoretical framework claims to fully encompass the range of plant—fungal interactions and their unique characteristics in nature^17^.

The rubber tree, *Hevea brasiliensis* (Willd.) Muell.-Arg., is the primary commercial source for natural rubber production^18^. Although *H*. *brasiliensis* is a native neotropical tree species of the Amazon biome and commercial rubber plantations in the Americas have largely failed due to South American Leaf Blight (SALB), a disease caused by the phytopathogenic fungus *Pseudocercospora ulei* (Henn.) Hora Júnior & Mizubuti^19^. Unlike plantation grown trees, incidence of disease among *H*. *brasiliensis* trees in their natural, undisturbed, habitat is low^19^. One of the hypotheses for this low incidence of SALB in native rubber trees is that they may harbor a protective endophytic mycobiota, which may be relatively abundant in plant tissue and directly acquired from their natural habitat^20,21^.

Given that fungal endophytes are mainly horizontally transmitted from the surrounding environment and that native habitat harbor protective fungal endophytes, we posit the following hypotheses: i) fungal endophytic communities significantly differ between study areas, ii) some of the most abundant fungal endophytes potentially represent mutualistic species that can be used as biological control agents of fungal diseases of rubber tree. In order to test these hypotheses, our study aimed to characterize the fungal endophytic communities of the foliar endosphere of native *Hevea brasiliensis* in two Eastern Amazonian pristine conservation units.

## Results

### Taxonomic composition

At the regional level, a total of 210 fungal endophytes isolates were obtained from the 240 leaflet fragments while, at the local level, 110 and 100 fungal endophytes isolates were obtained from ANP and CNF, respectively. A total of 46 putative species (OTUs) were retrieved, and these putative species are from two phyla, four classes, ten orders, 14 families and 21 (or 22) distinct genera. Two of the putative species (OTU 45 and 46) were not resolved to genus level and may be indeed a new genus (or genera). The majority of isolates were Ascomycota (95.71%) and only 4.28% were Basidiomycota (Table 1).

**Table 1:** List of putative species (OTU) identified and their complete taxonomic affiliation.

OTU No.	Putative species	Phylum	Class	Order	Family	Genus
01	*Arthrinium* sp. 1	Ascomycota	Sordariomycetes	Xylariales	Apiosporaceae	*Arthrinium*
02	*Aspergillus* sp. 1	Ascomycota	Eurotiomycetes	Eurotiales	Trichocomanaceae	*Aspergillus*
03	*Aspergillus* sp. 2	Ascomycota	Eurotiomycetes	Eurotiales	Trichocomanaceae	*Aspergillus*
04	*Clonostachys* sp. 1	Ascomycota	Sordariomycetes	Hypocreales	Bionectriaceae	*Clonostachys*
05	*Clonostachys* sp. 2	Ascomycota	Sordariomycetes	Hypocreales	Bionectriaceae	*Clonostachys*
06	*Clonostachys* sp. 3	Ascomycota	Sordariomycetes	Hypocreales	Bionectriaceae	*Clonostachys*
07	*Clonostachys* sp. 4	Ascomycota	Sordariomycetes	Hypocreales	Bionectriaceae	*Clonostachys*
08	*Colletotrichum* sp. 1	Ascomycota	Sordariomycetes	Glomerellales	Glomerellaceae	*Colletotrichum*
09	*Colletotrichum* sp. 2	Ascomycota	Sordariomycetes	Glomerellales	Glomerellaceae	*Colletotrichum*
10	*Colletotrichum* sp. 3	Ascomycota	Sordariomycetes	Glomerellales	Glomerellaceae	*Colletotrichum*
11	*Colletotrichum* sp. 4	Ascomycota	Sordariomycetes	Glomerellales	Glomerellaceae	*Colletotrichum*
12	*Cophinforma* sp. 1	Ascomycota	Dothideomycetes	Botryosphaeriales	Botryosphaeriaceae	*Cophinforma*
13	*Coriolopsis* sp. 1	Basidiomycota	Agaricomycetes	Polyporales	Polyporaceae	*Coriolopsis*
14	*Daldinia* sp. 1	Ascomycota	Sordariomycetes	Xylariales	Hypoxylaceae	*Daldinia*
15	*Diaporthe* sp. 1	Ascomycota	Sordariomycetes	Diaporthales	Diaportaceae	*Diaporthe*
16	*Diaporthe* sp. 2	Ascomycota	Sordariomycetes	Diaporthales	Diaportaceae	*Diaporthe*
17	*Diaporthe* sp. 3	Ascomycota	Sordariomycetes	Diaporthales	Diaportaceae	*Diaporthe*
18	*Diaporthe* sp. 4	Ascomycota	Sordariomycetes	Diaporthales	Diaportaceae	*Diaporthe*
19	*Entonaema* sp. 1	Ascomycota	Sordariomycetes	Xylariales	Hypoxylaceae	*Entonaema*
20	*Entonaema* sp. 3	Ascomycota	Sordariomycetes	Xylariales	Hypoxylaceae	*Entonaema*
21	*Hypoxylon* sp. 1	Ascomycota	Sordariomycetes	Xylariales	Hypoxylaceae	*Hypoxylon*
22	*Hypoxylon* sp. 2	Ascomycota	Sordariomycetes	Xylariales	Hypoxylaceae	*Hypoxylon*
23	*Lasiodiplodia* sp. 1	Ascomycota	Dothideomycetes	Botryosphaeriales	Botryosphaeriaceae	*Lasiodiplodia*
24	*Lasiodiplodia* sp. 2	Ascomycota	Dothideomycetes	Botryosphaeriales	Botryosphaeriaceae	*Lasiodiplodia*
25	*Lasiodiplodia* sp. 3	Ascomycota	Dothideomycetes	Botryosphaeriales	Botryosphaeriaceae	*Lasiodiplodia*
26	*Muscodor* sp. 1	Ascomycota	Sordariomycetes	Xylariales	Xylariaceae	*Muscodor*
27	*Nectria* sp. 1	Ascomycota	Sordariomycetes	Hypocreales	Nectriaceae	*Nectria*
28	*Nemania* sp. 1	Ascomycota	Sordariomycetes	Xylariales	Xylariaceae	*Nemania*
29	*Neofusicoccum* sp. 1	Ascomycota	Dothideomycetes	Botryosphaeriales	Botryosphaeriaceae	*Neofusicoccum*
30	*Neopestalotiopsis* sp. 1	Ascomycota	Sordariomycetes	Amphisphaeriales	Pestalotiopsidaceae	*Neopestalotiopsis*
31	*Penicillium* sp. 1	Ascomycota	Eurotiomycetes	Eurotiales	Trichocomanaceae	*Penicillium*
32	*Peniophora* sp. 1	Basidiomycota	Agaricomycetes	Russulales	Peniophoraceae	*Peniophora*
33	*Peniophora* sp. 2	Basidiomycota	Agaricomycetes	Russulales	Peniophoraceae	*Peniophora*
34	*Peniophora* sp. 3	Basidiomycota	Agaricomycetes	Russulales	Peniophoraceae	*Peniophora*
35	*Pleurostoma* sp. 1	Ascomycota	Sordariomycetes	Calosphaeriales	Pleurostomataceae	*Pleurostoma*
36	*Entonaema* sp. 2	Ascomycota	Sordariomycetes	Xylariales	Hypoxylaceae	*Entonaema*
37	*Pseudofusicoccum* sp. 1	Ascomycota	Dothideomycetes	Botryosphaeriales	Botryosphaeriaceae	*Pseudofusicoccum*
38	*Trichoderma* sp. 1	Ascomycota	Sordariomycetes	Hypocreales	Hypocreaceae	*Trichoderma*
39	*Trichoderma* sp. 2	Ascomycota	Sordariomycetes	Hypocreales	Hypocreaceae	*Trichoderma*
40	*Trichoderma* sp. 3	Ascomycota	Sordariomycetes	Hypocreales	Hypocreaceae	*Trichoderma*
41	*Trichoderma* sp. 4	Ascomycota	Sordariomycetes	Hypocreales	Hypocreaceae	*Trichoderma*
42	*Xylaria* sp. 1	Ascomycota	Sordariomycetes	Xylariales	Xylariaceae	*Xylaria*
43	*Xylaria* sp. 2	Ascomycota	Sordariomycetes	Xylariales	Xylariaceae	*Xylaria*
44	*Xylaria* sp. 3	Ascomycota	Sordariomycetes	Xylariales	Xylariaceae	*Xylaria*
45	Xylariaceae sp. 1	Ascomycota	Sordariomycetes	Xylariales	Xylariaceae	Unknown
46	Xylariaceae sp. 2	Ascomycota	Sordariomycetes	Xylariales	Xylariaceae	Unknown

Most of the Ascomycota isolates were Sordariomycetes (85.23%), and only 8.57% comprised Dothideomycetes, with an even lower value for Eurotiomycetes (1.90%). The most abundant order belonging to Sordariomycetes were Glomerellales (55.90%), followed by Hypocreales (27.93%), Xylariales (11.12%), Diaporthales (13.04%), and Calosphaeriales and Amphisphaeriales representing only 0.56% each. The other two classes of Ascomycota, Dothidiomycetes and Eurotiomycetes, were both represented by only one order (and each order by only one family): Botryospheriales (Botryospheriaceae) and Eurotiales (Trichocomaceae). The Basidiomycota putative species were all of one single class, Agaricomycetes, distributed in two orders, Russulales (88.89%) and Polyporales (11.11%), which is both represented by a single family, Peniophoraceae and Polyporaceae, respectively (Table 1).

**Figure 1:**
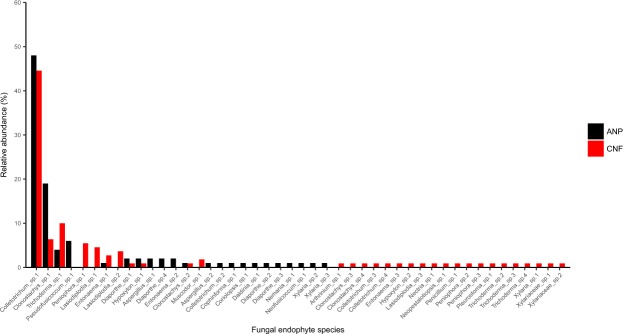
Relative abundance of the fungal endophyte species obtained from each study area.

The most abundant genera were *Colletotrichum* (47.62%), followed by *Clonostachys* (14.29%), *Trichoderma* (8.57%), *Lasiodiplodia* (4.76%), *Peniophora* (3.81%), which together comprised 69.16% of all the isolates (Fig. 1). The remaining 20.95% of the isolates belonged to 18 genera (Table 1). There were 22 putative species (OTUs) recorded from Anavilhanas National Park (ANP) and 31 from Caxiuanã National Forest (CNF). Seven putative species, *Clonostachys* sp. 1, *Clonostachys* sp. 2, *Colletotrichum* sp. 1, *Diaporthe* sp. 1, *Entonaema* sp. 1, *Hypoxylon* sp. 1, *Trichoderma* sp. 1, which represent collectively only 15.22% of the total number, were found in both locations. The great majority of putative species (84.78%) are either exclusively of ANP (32.60%) or, even more, of CNF (52.18%). *Colletotrichum* sp. 1 was the only putative species obtained from all host trees in both study areas and was recovered with a relative abundance of 48.0% in ANP and 44.5% in CNF (Fig. 1).

### Community diversity

**Figure 2:**
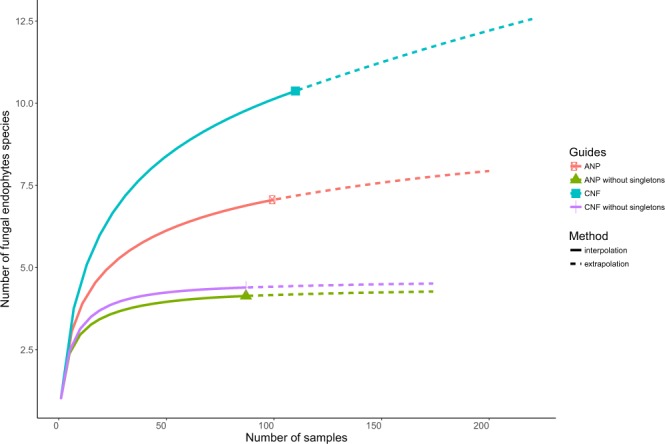
Rarefaction curve of the number of fungal endophytes species against the number of samples in each study area.

Species accumulation curves did not reach asymptote for either study area, indicating that the total number of expected species was not captured. Richness was significantly different for both study areas. Sampling effort based on Chao2 estimator was 56.6% and 10.5% for ANP and CNF, respectively (Table 2), and species accumulation curves neared asymptote when singletons were excluded (Fig. 2). Normalized Shannon index and evenness were lower in ANP than in CNF (Table 2). Simpson index was similarly high for both collection sites, indicating that communities are dominated by few fungal species: *Colletotrichum* sp. 1 and *Clonostachys* sp. 1 represented 67% of the total abundance in ANP while *Colletotrichum* sp. 1 and *Trichoderma* sp. 1 comprised 54.6% in CNF.

**Table 2:** Description of the study sites and diversity indexes of the fungal endophytes associated to *Hevea brasiliensis*.

Park	Geographical coordinates	Total number of isolates	Colonization Rate (%)	Richness	Shannon	Shannon normalized	Simpson	Simpson normalized	Evenness	Chao2
Anavilhanas		100	61.67	22	1.96	7.10	0.73	3.64	0.17	38.90
Ind 1	03°00.12.06″S 060°39′01″W	25	80.0							
Ind 2	03°00′12.00″S 060°39′03″W	23	66.7							
Ind 3	02°59′52.26″S 060°29′09″W	21	60.0							
Ind 4	02°59′52.02″S 060°30′01″W	31	90.0							
Caxiuanã		110	85.83	31	2.34	10.38	0.79	4.51	0.15	295.5
Ind 1	01°45′59.9″S 51°24′17.2″W	27	86.7							
Ind 2	01°45′59.8″S 51°24′17.0″W	27	90.0							
Ind 3	01°45′59.5″S 51°24′17.5″W	29	96.7							
Ind 4	01°46′00″S 51°24′16.8″W	27	86.7							

Colonization frequency is the percentage of leaf fragments from which at least one fungal culture was isolated.

**Figure 3:**
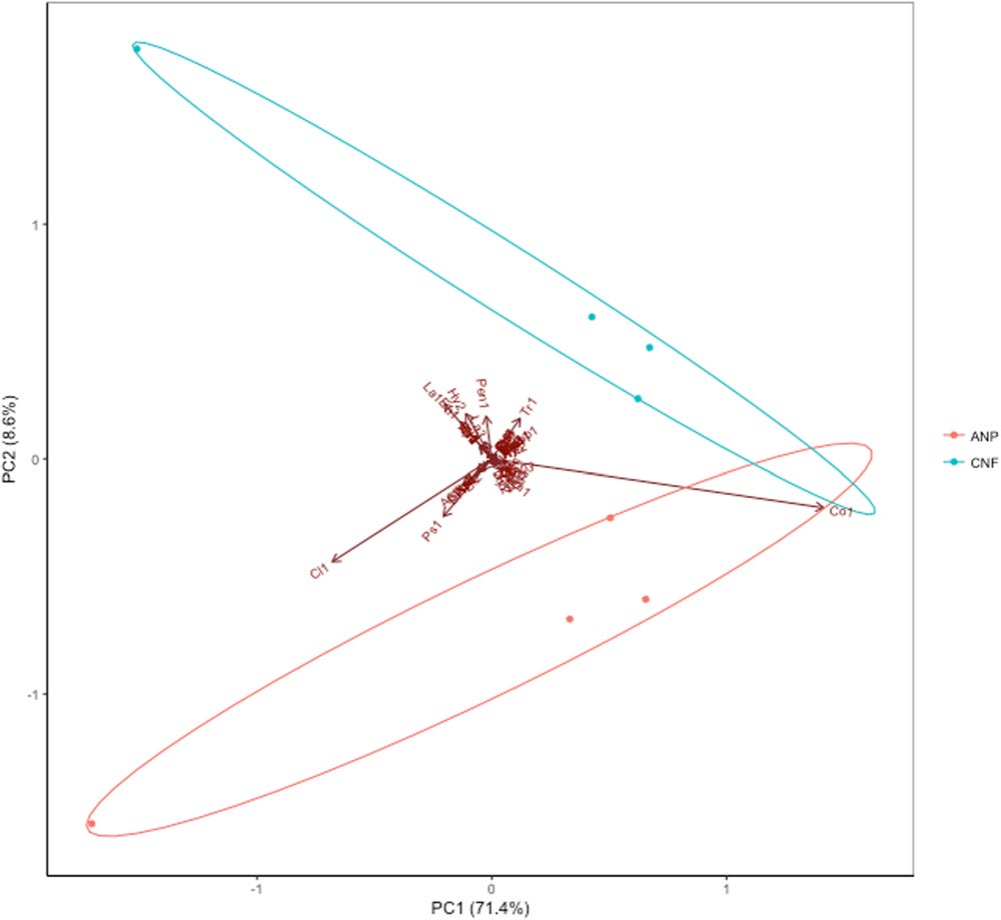
Principal component (PCA) plot of fungal endophyte associated to foliar tissues of *Hevea brasiliensis*.

The first two components in the PCA explained 80% of the variation, and there was a clear separation among the samples from the two study areas (Fig. 3). PERMDISP analysis showed that the community dissimilarity variances in each study area (regional scale - F = 0.002, *P* = 0.96 with singletons, and F = 0.01, *P* = 0.92, without singletons) and among individual trees (local scale - F = 1.19, *P* = 0.31 with singletons, F = 2.17, *P* = 0.03, without singletons) did not significantly differ, which corroborates the previous results showing that two putative species dominate the communities, regardless considering or not the singletons. The only exception was for the individual trees when the singletons were removed. The distance decay analysis also corroborated this finding (Rate of DD = 0.05, *P* value = 2.58 10^8^). PERMANOVA analyses showed that the community composition (presence and absence) of the study areas (regional scale - r^2^ = 0.02, *P* = 0.005 with singletons, r^2^ = 0.02, *P* = 0.01 without singletons) and of the individual trees (local scale - r^2^ = 0.021, *P* = 0.009, with singletons and r^2^ = 0.03, *P* = 0.001 without singletons) were significantly different, regardless considering or not the singletons.

**Figure 4:**
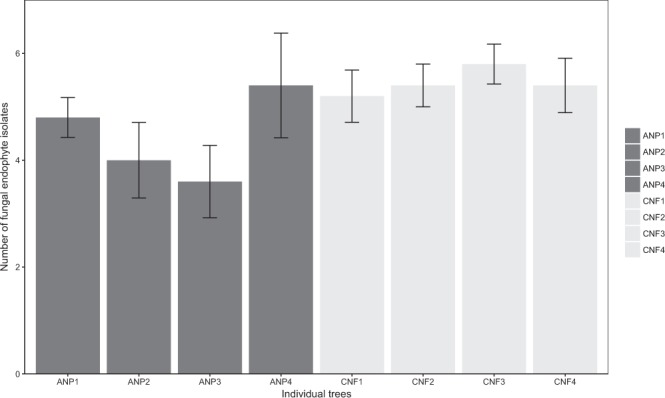
Mean (± standard error) number of fungal endophytes isolated from the different individual trees. An: Anavilhanas, Cx: Caxiuanã.

Colonization Rate (CR) was similar in CNF and ANP (Table 2) (Friedman test, Χ = 2.25, *P* = 0.14), and the mean number of isolates was not statistically different among the individual trees in both study areas and between them (Fig. 4). There was a highly significant negative correlation between the two most abundant taxa in both study areas: *Colletotrichum* sp. 1 and *Clonostachys* sp. 1 in ANP; *Colletotrichum* sp. 1 and *Trichoderma* sp. 1 in CNF. In 98.08% of the sampling units (leaflet fragments) in ANP, when one of the taxa occurred (regardless if one or more than one isolate was retrieved), the other necessarily did not occur (*P* = 0.01). This very high negative correlation reached the maximum in CNF: in 100% of the sampling units (leaflet fragments) when one of the taxa occurred (regardless if one or more than one isolate was retrieved, too), the other necessarily did not occur (*P* = 0.01).

ALR models converged to the hierarchical levels described in Table 3. The leaflet fragments were considered in the mean structure, and the values varied according to the taxonomic level. Statistically significant differences varied depending on the taxonomic category evaluated. At the dependence structure, the odds ratio was statistically significant at individual (tree) and intra-individual (leaflet) scales only for Bionectriaceae, *Clonostachys*. Distances among trees were statistically significant only for Glomerellales, Glomerelaceae and *Colletotrichum*.

**Table 3:** Alternating Logistic Regression (ALR) statistical analyses considering the fungal endophyte levels of order and class.

Mean structure	Sordariomycetes	Glomerellales Glomerelaceae Colletotrichum	Hypocreales	Bionectriaceae Clonostachys	Hypocreaceae Trichoderma
	O.R.	O.R.	O.R.	O.R.	
Intercept (β)	0.90 (0.78–1.03)	0.43 (0.42–0.45)*	0.15 (0.12–0.19)*	0.06 (0.05–0.07)*	0.06 (0.02–0.22)*
Fr = A	2.58 (1.53–4.34)*	1.27 (0.95–1.69)	1.48 (1.38–1.59)*	1.76 (1.36–2.27)*	1.00 (1.00–1.00)
Fr = B	1.84 (1.36–2.50)*	2.38 (1.85–3.06)*	1.23 (0.91–1.67)	1.37 (0.70–2.69)	1.00 (0.26–3.82)
Fr = D	2.91 (2.13–3.98)*	1.75 (1.49–2.07)*	2.64 (1.17–5.94)*	2.60 (1.09–6.18)*	2.70 (0.95–7.69)
Fr = E	2.58 (1.10–6.03)*	1.75 (1.18–2.62)*	1.48 (1.38–1.59)*	1.37 (0.70–2.69)	1.54 (0.64–3.71)
Fr = F	2.05 (1.28–3.28)*	1.42 (1.20–1.67)*	2.02 (1.54–2.66)*	1.76 (1.36–2.27)*	1.54 (1.47–1.61)*
**Dependence structure**					
Intercept (Site collection α_1_)	1.04 (0.98–1.09)	0.82 (0.72–0.94)	0.87 (0.66–1.13)	0.62 (0.33–1.15)	1.12 (0.07–18.95)
Distance (α_2_)	1.07 (0.94–1.22)	1.90 (1.55–2.33)*	1.09 (0.75–1.58)	2.95 (0.94–9.29)	0.63 (0.15–2.65)
Individual tree (α_3_)	1.37 (0.79–2.40)	1.66 (0.92–3.00)	2.27 (0.69–7.48)	2.60 (1.12–6.06)*	6.63 (0.07–6.00 10^2^)
Leaf (α_4_)	1.00 (1.00–1.00)	1.00 (1.00–1.00)	1.00 (1.00–1.00)	1.00 (1.00–1.00)*	1.00 (1.00–1.00 10^6^)

Significant values in bold: *P < 0.05.

The values inside the brackets correspond to the inferior limit and superior limit. **Fr:** Leaf fragment.

## Discussion

We performed a multiscale study of culturable fungal communities of the foliar endosphere of a native hyperdiverse tree (*Hevea brasiliensis*) in the Eastern Amazon. A high number of leaflet fragments showed at least one fungal endophyte isolate (CR- ANP: 61.7 and CR-CNF: 85.8), and similar values were obtained for *Hevea brasiliensis* (CR: 72 in Western Amazon^19^, and from other tropical plant hosts, such as *Macrosolen cochinchinensis* (Loranthaceae) in southern China^22^, from *Theobroma cacao* (Malvaceae)^23^ and from *Heisteria concinna* (Olacaceae) and *Ouratea lucens* (Ochnaceae) in Panama^4^.

According to the species accumulation curves, the sampling effort was not sufficient to adequately capture the fungal endophyte richness (Fig. 2), a pattern frequently found in community ecology studies of fungal endophytes in tropical environments^4,12,20^. Most of the putative species were single occurrences (singletons), and similar values was previously found by Gazis & Chaverri^20^, who studied the same host tree species in Western Amazon, as well as in many tropical trees in Barro Colorado Island in Panamá^2^. The singletons may represent rare species^2,24^, and probably, the increase in the sampling effort would capture mainly these species^22^. After removing the singletons, the accumulation curves approximated to an asymptote (Fig. 2). The high number of singletons also reflected the low evenness obtained from both study areas, and, when they were removed, there was a sharp increase in the evenness. Therefore, there are many rare taxa in native *Hevea brasiliensis* foliar endosphere, which is in complete accordance with the prediction of the dual-axis framework for examining fungal endophyte diversity^17^. These rare taxa are horizontally transmitted,and they do not exhibit host colonization preference and, thus, adopt a more beneficial strategy to be a rare colonizer of many host species^17^.

Methods based on culture are influenced by the composition of the culture media, the physiological adaptations of the fungi, and the sampling procedures^25^, and all of these factors could influence the richness and abundance of endophytic fungi recovered. In fact, the Shannon index was similar to those obtained for the tropical tree hosts *Myrceugenia ovata* and *Eugenia neomyrtifolia* in Vaz *et al*.^12^, who used exactly the same sampling procedure. However, the values were lower than those from *Hevea brasiliensis* in Western Amazon^20^, where three leaflets fragments were sampled per tree and CMA (Corn meal agar) was used with a nutritional supplement (2% dextrose). These differences in the culturable methodologies could explain the discrepancies in the diversity indices values obtained in the present work when compared to other similar studies.

The beta diversity of fungal endophyte communities associated to *Hevea brasiliensis* was explored at the regional scale, over distances of approximately 1,000 Km. The non-systemic fungal endophytes are horizontally transmitted by hyphal fragmentation and/or by spores from plant to plant^4,5,26^. Thus, the mycobiota surrounding the host trees in each study area are probably responsible for the fungal endophytes that reach and colonize the tree hosts, regardless if the hosts are from the same tree species^27^. Although the fungal endophytes are capable of dispersing and reaching host trees, they are subjected to environmental variables, which act as filters and select those better adapted to local conditions^14^. In our work, the environmental variables were quite similar between the two study areas and, thus, the main factor contributing to the community composition differences (PERMANOVA) were, most probably, the fungal source at local scale since the tree species diversity is distinct between the two regions^28,29^.

*Colletotrichum* sp. 1 was the most abundant species in both study areas. The genus *Colletotrichum* is a very speciose genus with 805 putative species^30^ (Mycobank, access in 12 Jun 2018) and comprises hemibiotrophic phytopathogens of major importance, causing diseases of a wide variety of woody and herbaceous plants, primarily with tropical and subtropical distribution^31^. *Colletotrichum* species are primarily described as causing anthracnose diseases, mainly necrotic lesions on leaves, besides on flowers and fruits^32^.

All the isolates identified as *Colletotrichum* sp. 1 in both collection sites correspond to the UNITE Species Hypothesis SH103151.07FU^33^. This SH group comprises 1,086 records of *Colletotrichum gloeosporioides* species complex from all over the world, including 53 records from *Hevea* spp. (38 from *Hevea brasiliensis*) with genetic distances equal or less than 1% compared to ANP and CNF *Colletotrichum* sp. 1 isolates. The gloeosporioides species complex is a collective of *C*. *gloeosporioides* and 37 closely related species that mainly encompass plant pathogens, with some species also isolated as endophytes^34^. Furthermore, *Colletotrichum gloeosporioides* species complex has already been recorded associated to more than 1,000 distinct plant species^35^ (ARS Fungus-Host Database, access in 12 Jun 2018). One of the five major leaf diseases that can cause damage to *Hevea brasiliensis* in different countries is the *Colletotrichum* leaf disease, which is caused precisely by a *Colletotrichum gloeosporioides*^36^, which appears in older literature as *Colletotrichum heveae*^37^ or *Colletotrichum gloeosporioides f*. *heveae*^38^.

*Colletotrichum* appears as the most abundant genus or one of the most abundant genera in different fungal endophyte culture-based studies globally, especially in tropical tree hosts^39^. It was the most abundant genus obtained from plantations of *H*. *brasiliensis* (Euphorbiaceae)^20^ and *Musa acuminata* (Musaceae) in China^40^ as well as from native *Manilkara bidentata* (Sapotaceae) in Guyana^31^, *Myrceugenia ovata* (Myrtaceae) in Brazil^12^, and *Plumeria rubra* (Apocynaceae) in India^41^. *Colletotrichum* was also the third most abundant fungal endophyte from native *H*. *brasiliensis* in Peru^37^ and *Tectona grandis* (Verbenaceae) in India^42^. According to the dual-axis framework for examining fungal endophyte diversity^17^, fungal endophyte genera that contain many pathogenic members often show very broad geographic distribution and low host specificity: this is exactly the case of *Colletotrichum*, suggesting that our putative species *Colletotrichum* sp. 1, our most abundant endophyte in both study areas, may be a latent pathogen^43^.

The distance between host tree species inside each study area was statistically significant (Table 3) to explain the *Colletotrichum* sp. 1 distribution, indicating that there is a higher odds ratio of finding one isolate of this species when the host tree species are near each other, regardless if these fungal propagules are from the same leaflet or individual host tree. Moreover, *Colletotrichum* sp. 1 did not exhibit host preference by *Hevea brasiliensis* trees evaluated in this study, which corroborates the idea that this species is widespread and ubiquitous at regional scale^39,43^. On the other hand, we found statistically significant evidence for host colonization preference by two different hypocrealean endophytes. The second most abundant genera obtained from the host trees in ANP and CNF was *Clonostachys* sp. 1 (Hypocreales, Bionectriaceae) and *Trichoderma* sp. 1 (Hypocreales, Hypocreaceae), respectively.

*Clonostachys* sp. 1 was the second most abundant species in ANP host trees and showed an association at the leaflet and individual host tree levels (Table 3). Therefore, the odds ratio of its dispersal is greater inside the same leaflet/individual than outside. *Clonostachys* is the anamorph of the genus *Bionectria* and encompass 76 putative species^30^ (Mycobank, access in 12 Jun 2018). Besides other life-styles, species of *Clonostachys* include destructive mycoparasites, some of which are used as biocontrol agents of fungal plant pathogens^44^. All the isolates identified as *Clonostachys* sp. 1 correspond to the UNITE Species Hypothesis SH182678.07FU^33^. This SH group comprises 294 records of *Clonostachys rosea* from distinct countries. *Clonostachys rosea* is a necrotrophic mycoparasitic fungus, used as a biological control agent of many phytopathogenic fungi, such as *Alternaria* spp., *Bipolaris sorokiniana*, *Botrytis cinerea*, *Fusarium culmorum*, *Fusarium graminearum* and *Sclerotinia sclerotiorum*^45^ and *Moniliophthora perniciosa*^46^.

*Trichoderma* sp. 1 was the second most abundant genus obtained from the host trees in CNF. *Trichoderma* is also a common fungal endophyte genus and was one of the most abundant from native *Hevea* spp. in Western Amazonia^20,47^. *Trichoderma* is the anamorph of the genus *Hypocrea* and comprises 367 putative species^30^ (Mycobank, access in 12 Jun 2018). *Trichoderma* spp. are among the most frequently isolated soil fungi and present in plant root ecosystems^48^. These fungi are avirulent plant symbionts, and parasites of many phytopathogenic fungi, thus protecting plants from diseases. *Trichoderma* species are among the most studied fungal Biological Control Agents (BCAs) and commercially marketed as biopesticides^49^. All the isolates identified as *Trichoderma* sp. 1 correspond to the UNITE Species Hypothesis SH190868.07FU^33^. This SH group comprises 2,161 records of *Trichoderma harzianum* from distinct countries all over the world. The *Trichoderma harzianum* species complex is a collective of 14 closely related species that mainly encompass mycoparasites or fungicoles with a long history in agricultural applications, especially those related BCA of phytopathogenic fungi^47^. Therefore, *Clonostachys* and *Trichoderma* have been long considered effective biocontrol agents against diverse fungal phytopathogens in nature and agroecosystems^46,50^.

The generalist fungal endophytes exhibit fast growth, the hyphal extension increases their competition by resources, and their foliar colonized area is larger, which make them more easily isolated in culture using the standard media^16^. In our work, we suggested that the fungal endophyte *Colletotrichum* sp. 1 is widespread in the environment and reaches and colonizes the foliar tissue of all plant hosts.

The traits related to the environment where the organisms establish in communities are evolutionary conserved, as a result, related species may function ecologically similar^15^. *Clonostachys* sp. 1 and *Trichoderma* sp. 1 are from the same order (Hypocreales), exhibited a lower abundance compared to the most abundant taxon, and, most importantly, are mycoparasitic or fungicoles, suggesting that these two different taxa share the same ecological function in the foliar tissues of the host. Furthermore, previous works reported that slower growth fungal endophytes are able to produce secondary metabolites that inhibit possible pathogens^46^. Although we had not evaluated the antibiosis potential of *Clonostachys* sp. 1 and *Trichoderma* sp. 1 against *Colletotrichum* sp. 1 in *in vitro* antagonistic bioassays, they would probably be potential candidates as biological control agents of *H*. *brasiliensis* fungal pathogens by secondary metabolite production.

## Conclusions

Our study showed that the fungal endophyte community composition of the foliar endosphere of native rubber trees in Eastern Amazon significantly differed between the study areas. Nevertheless, the endophytomes exhibited two general features in common, which are in complete accordance with the dual-axis framework for decoding fungal endophyte diversity^23^: They are dominated by very few core taxa and highly skewed toward infrequently isolated rare taxa. These core taxa comprised the most abundant putative species, *Colletotrichum* sp. 1, a probable latent pathogen, and the phylogenetically related hypocrealean putative species, *Clonostachys* sp. 1 and *Trichoderma* sp. 1, which are mycoparasites that potentially represent mutualistic species performing the same ecological function in ANP and CNF, respectively. The next steps in our research program will be to test both *in vitro* and *in planta* the antagonism of *Clonostachys* sp. 1 and *Trichoderma* sp. 1 against *Colletotrichum* sp. 1 and *Hevea brasiliensis* phytopathogenic strains, *Colletotrichum gloeosporioides* and *Pseudocercospora ulei*, causal agents of *Colletotrichum* leaf disease and SALB, respectively.

## Methods

### Study areas

Fieldwork was conducted in two protected areas in Eastern Amazonia, distant each other approximately 1000 Km: (i) Caxiuanã National Forest (CNF) and (ii) Anavilhanas National Park (ANP). CNF is the eastern limit of the natural geographic distribution of *Hevea brasiliensis*, whereas ANP is in the center of origin of the genus *Hevea*^51^. Fieldwork permits were obtained from the Brazilian Ministry of Environment (MMA) under the access code SISBIO 42316-2.

CNF is situated in the state of Pará, Brazil (Lat. 01°37′S – 02°15′S; Long. 51°19′W – 51°58′W), with an altitude ranging from 0–80 m and a total area of 330,000 ha. The majority of the landscape is composed of non-flooded forests (85%) and seasonally or permanently flooded forests (15%). The regional climate is classified as Am in the Köppen climate classification system, with a mean annual temperature of 25.9 °C, a mean annual rainfall of 2011 mm, mean annual air relative humidity of 83%, and a short dry season^29^.

ANP is situated in the state of Amazonas, Brazil (Lat. 02°03′S – 03°02′S; Long. 60°22′W – 61°12′W), with an altitude ranging from 40–75 m and a total area of 350,018 ha. The majority of the landscape is composed of non-flooded forests (70%) and seasonally or permanently flooded forests (30%). The regional climate is classified as Af in the Köppen climate classification system, with a mean annual temperature of 26 °C, a mean annual rainfall of 2286 mm, mean annual air relative humidity of 83%, without a dry season^29^.

### Fungal endophyte isolation

**Figure 5:**
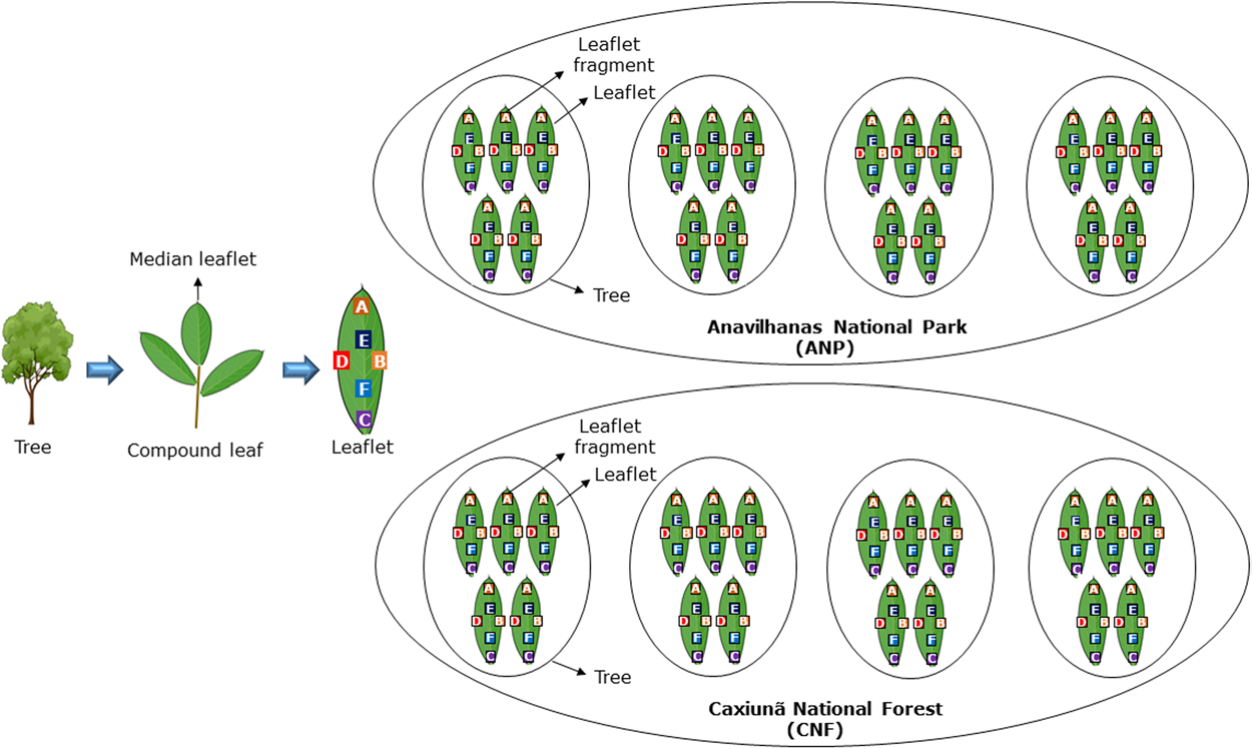
Multiscale sampling design for investigating fungal endophyte communities of native rubber trees in two pristine areas in Eastern Amazon.

Four adult individuals of *Hevea brasiliensis* were randomly selected from each study area. Five visually healthy compound leaves (three leaflets per leaf) with homogeneous green coloration, and without any wilting or necrotic lesions were sampled from each one of the four adult individual trees. All leaves were at D developmental stage (mature leaves)^19^ and were collected at 2.5–3.5 m height. Geographical coordinates were recorded for each sampled tree using a handheld GPS unit (GPSmap 62 s, Garmin Inc., Schaffhausen, Switzerland). All the compound leaves were maintained in individualized sterile plastic bags and the fungal isolation was performed in the maximum period of time around 3–4 hours, the samples were not maintained cool The median leaflet from each compound leaf was detached and rinsed under running tap water to remove dirt and debris. After which, the leaflet was surface-sterilized via sequential dipping in 70% ethanol (1 min), 2% sodium hypochlorite (3 min), and sterile distilled water (2 min). Six fragments (approximately 5 mm^2^) were excised from each leaflet in specific positions: one from the base near petiole, two from the middle vein, one from the left margin, one from the right margin and from the tip^26^ (6 fragments/leaflet; 30 fragments/individual tree; 120 fragments/site; 240 overall – Fig. 5).

The leaflet fragments were plated onto malt extract agar (MEA) (Acumedia, EUA), supplemented with chloramphenicol (Himedia, India) (100 mg/L^−1^) and Rose Bengal (Vetec, Brazil) (30 mg/L^−1^) to inhibit the bacterial contamination and rapidly growing fungi^52^. The plates were incubated at room temperature, in the dark, for up to 60 days. The effectiveness of the surface sterilization was tested by plating 100 µl of the water used in the last step and incubated under the same conditions as the media plates. Emerging fungal colonies were purified on MEA without bactericidal or bacteriostatic compounds. The isolated were preserved in sterile distilled water at room temperature^53^. Vouchers (testimony specimens) were preserved in sterile distilled water and stored in CCMB (Culture Collection of Microorganisms of Bahia) (UEFS - Brazil) under the accessions CCMB660 to CCMB870.

### DNA barcoding of endophytic fungi

Mycelial mats were ground using liquid nitrogen. Ground up mycelia (~150 mg) was then placed into a 1.5 mL tube containing 600 µL of extraction buffer (Tris-HCl pH 9, 0.005 M, 1% NaCl 0.1 SDS, 3% β-mercaptoethanol, and 3% polyvinyl-pyrrolidone - PVP) and Proteinase K 50 µg/ml. Samples were incubated at 60 °C for 60 minutes, after which 800 µL of chloroform-isoamyl alcohol (24:1) was added to each tube. Samples were then incubated on ice for 30 minutes. After incubation period, DNA was precipitated according to De Hoog *et al*.^54^. DNA was diluted to 1:100 for amplification using Polymerase chain reaction (PCR). The internal transcribed spacer (ITS) regions of rRNA gene were amplified using ITS4 (5′-TCCTCCGCTTATTGATATGC-3′) and ITS5 (5′-GGAAGTAAAAGTCGTAACAAGG-3′)^55^. PCR was performed according to Vaz *et al*.^56^ with modifications: it was added 10 uL of Betaine 1 M, 1 μL of Dimethyl sulfoxide (DMSO) 50 and 1.5 μL of Bovine Serum Albumin (BSA) 0,031 μg/μL in PCR reaction. Successfully amplified PCR products were purified using an ethanol/ethylenediaminetetraacetic acid 125 mM precipitation protocol and the sequencing reactions were performed at Myleus Biotechnology (www.myleus.com, Belo Horizonte, Brazil) on an ABI 3130 automated sequencer (Applied Biosystems, Life Technologies Q7, CA, USA) Sequences were edited using Geneious (version 9.1.6)^57^.

Full-length ITS sequences (450–650 bp) of fungal isolates were aligned with MAFFT 7.305^58^ in Geneious (version 9.1.6)^57^. Default parameters were selected, along with the adjust direction option. Subsequently, sequences were clustered into operational taxonomic units (OTUs) using the furthest neighbor method in MOTHUR v. 1.36.1^59^ with a 99% similarity criterion^60^. One representative sequence from each putative species was chosen for OTU taxonomic classification. BLASTn^61^ was used to compare sequences against the GenBank nucleotide database excluding uncultured/environmental sample sequences. GenBank sequences of the matched taxa for all OTUs were collected. When possible, well-curated published sequences with voucher/culture collection numbers were selected. Representative sequences from our study were then aligned with GenBank sequences and clustered into OTUs as previously described. The percent similarity at which all the GenBank representative sequences clustered together exclusively, with no other representative sequences from other genera were clustered with them, was considered the genus limit for the OTU. The final edited sequences were deposited in NCBI Genbank under accessions MG490657-MG490860; MG800849-MG800854.

### Analysis of ecological data

The species diversity was measured through species richness, abundance and diversity index, which combine both richness and abundance^62^. The diversity was estimated using the Shannon (H’) (*H* = *−Σ ni/n ln* (*ni/n*) and Simpson indices (*D* = 1*−Σ (ni/n*)^2^*)*, where *ni* is the number of individuals of the taxon *i*, and *n* is the total number of individuals. To find the effective number of species these both indices were modified: Shannon normalized (*exp (H’))* Simpson normalized (*1/(1 − D*))^63^. The homogeneity of abundances (evenness) was measured using the Pielou formula (*H’/H’max*)^64^. The Chao2 (S_chao2_ = S_obs_ + (m − 1/m) (q_1_(q_1_ − 1)/2(q_2_ + 1)), where *S*_*obs*_ is the total observed number of species, *m* is the number of samples, q_1_ is the number of uniques (species that occur in one sample), q_2_ is the number of duplicates (species that occur in two samples)^65^. For statistical analysis, each individual fragment was considered a sample unit and a total of 240 sample units were evaluated. Rarefaction curves was performed to indicate if the number of sampling units was sufficient to wholly capture the diversity and to extrapolate the species richness using iNEXT package^66,67^.

The data was evaluated at four different spatial scales: regional, local, individual (tree), and intra-individual (leaflet). The geographic distance among was measured for regional (101–5,000 km) and local (0–100 km) scales. There was no possible to determine the geographic distance to the other because individual scale correspond to the samples collected from the same individual host tree and the intra-individual the samples took from different parts from the same leaf. Principal Component Analysis (PCA) was conducted to visualize the trend and grouping to the fungal endophytes at regional and local scales using the vegan package^68^. The rate of distance decay of the fungal endophyte communities was calculated according to Nekola & White^69^, with the assumption that community similarities decrease with increasing geographical distance. A randomization procedure with 1,000 iterations was implemented to test whether the slope of the distance decay curve was significantly different from zero.

In order to test the heterogeneity of the community between the study areas (local scale) and individuals host trees (individual scale), the permutational test of multivariate dispersion (PERMDISP) was used. Leaflet fragments that did not exhibit fungal endophyte growth were removed from the datasets prior to PERMANOVA and PERMIDISP analyses. Therefore, the size of the Anavilhanas dataset (67 leaflet fragments) differed from the Caxiuanã dataset (93 leaflet fragments).

Colonization rate was calculated using the following equation^70^:

$$\begin{array}{c}{\mathtt{CR}}\,({\mathtt{ \% }}){\mathtt{=}}{\mathtt{[}}\mathrm{Number}\,{\mathtt{of}}\,{\mathtt{leaflet}}\,{\mathtt{fragments}}\,{\mathtt{colonized}}\,{\mathtt{with}}\,\ge \,{\mathtt{1}}\,{\mathtt{isolate}}/{\mathtt{Total}}\,{\mathtt{number}}\\ \,{\mathtt{of}}\,{\mathtt{leaflet}}\,\mathrm{fragments}{\mathtt{]}}\ast {\mathtt{100}}{\mathtt{.}}\end{array}$$

The Friedman test, followed by the Tukey post hoc test, was used to determine whether the CR differences, either among host tree individuals (local scale) or among leaflets of an individual host tree (individual scale), were statistically significant^71^. In order to evaluate if there was any statistically significant association between the most abundant taxa, a correlation analysis was performed using the non-parametric Spearmann’s D (rank-order correlation coefficient) as the test criterion and 5% significance level using Hmisc package^72^.

In order to test the probability of re-encountering a particular fungal taxon within a leaflet fragment, leaflet, host tree, or study area, an Alternating Logistic Regression (ALR) was used^67^ (Lipsitz *et al*., 1991). The ALR models were analyzed at all fungal taxonomic levels for all genera obtained. The mean and structure was modeled with the marginal odds ratio^73^. A pairwise odds ratio significantly different from the null suggests the presence of fungal endophytes within a nesting level. The distance reflects the odds ratio of finding another fungal endophyte of the same taxonomic level previously found when comparing two individual host trees spaced one meter apart. Fungal endophyte singletons were not included in the modeling analysis. The ALR analysis was performed using the *ordgee* function from geepack package^74^. All analysis was carried out using the vegan package^75^ and the R script.
